# Effect of dietary patterns on mild cognitive impairment and dementia: a machine learning bibliometric and visualization analysis

**DOI:** 10.3389/fnut.2024.1378959

**Published:** 2024-05-13

**Authors:** Yan Lou, Xueping Chen, Le Zhao, Nan Xuc, Lijun Zhang, Wenyi Hu, Yongzhen Qiu

**Affiliations:** ^1^Haining Health School, Jiaxing, Zhejiang, China; ^2^Hangzhou Yanjiang Technology Co., Ltd., Hangzhou, Zhejiang, China; ^3^Quzhou College of Technology, Quzhou, Zhejiang, China; ^4^Hangzhou Lvkang Hospital Co., Ltd., Hangzhou, Zhejiang, China; ^5^China Jiliang University, Hangzhou, Zhejiang, China; ^6^Department of Cardiology, Lishui Municipal Central Hospital, Lishui, Zhejiang, China

**Keywords:** mild cognitive impairment, dementia, dietary, non-pharmacological intervention, bibliometrics, visualization analysis

## Abstract

**Objective:**

As a spectrum of neurodegenerative conditions, dementia presents a significant challenge to worldwide health. Mild cognitive impairment (MCI) is recognized as the intermediate stage between normal cognitive functioning and dementia. Studies highlight the significant impact of dietary patterns on the management of MCI and dementia. Currently, comprehensive research on dietary patterns specific to MCI and dementia is limited, but bibliometric analysis offers a method to pinpoint essential research directions.

**Methods:**

On November 18, 2023, a search was conducted in the Web of Science Core Collection (WoSCC) for publications on diet and MCI/dementia. Tools such as Rstudio, CiteSpace, and VOSviewer were employed to create a knowledge atlas. This atlas analyzed collaborations, reference co-citations, keyword patterns, and emerging trends.

**Results:**

The search yielded 1,493 publications on diet and MCI/dementia, indicating a growing interest despite fluctuations. Contributions came from 70 countries/regions and 410 organizations across 456 journals. The USA and China led in publication numbers, with significant contributions from Columbia University and Harvard Medical School. Top authors include Scarmeas Nikolaos, Morris Martha Clare, and Samieri Cecilia. The Ketogenic, Mediterranean, and MIND diets emerged as key dietary patterns for cognitive decline prevention, highlighting the role of genetic factors, especially ApoE polymorphisms, in cognitive deterioration.

**Conclusion:**

This study provides core countries, institutions, and authors in the field, and points out the development directions in the field. Future research directions in dietary for MCI and dementia will focus on: (1) the potential effects of the KD in alleviating oxidative stress and modulating gut microbiota in neurodegenerative diseases; (2) how diet influences cognitive health through patterns of ApoE and protein expression; (3) investigating the interactions between gut microbiota and brain function, known as the “gut-brain axis.”

## 1 Introduction

Dementia, a spectrum of neurodegenerative conditions including Alzheimer’s disease (AD)—the most prevalent cause in elderly adults—along with Vascular Dementia, Frontotemporal Lobar Degeneration, and Lewy Body dementia, poses a significant and growing global health challenge (1). Clinically characterized by global dementia manifestations such as memory impairment, aphasia, apraxia, et al., the etiology of which is still unknown (2, 3). Alzheimer’s accounts for 60–80% of cases, with the collective incidence expected to rise in parallel with the aging population, forecasting a surge from 57.4 million cases in 2019 to 152.8 million by 2050 (4, 5). Mild cognitive impairment (MCI) is the transitional phase between normal cognitive functioning and dementia, people affected by MCI are at greater risk of dementia (6). Depending on the sample studied and the follow-up duration, conversion rates from MCI to dementia range from 5–20% (7). Health care will be burdened by these rapidly growing numbers, which will have a significant impact on society (8).

Although clinical symptoms such as memory loss and language difficulties are well-characterized, the etiology of dementia remains elusive, and disease-modifying treatments are notably absent. Current pharmacological interventions for MCI and dementia are few and often accompanied by substantial side effects (9), thereby underscoring the importance of prevention. The World Health Organization (WHO) recommends that governments take global actions against dementia and cognitive decline, focusing on prevention, disease-modifying therapies, and improving health care (10). A large number of studies have confirmed that diet may can control cognitive decline in many aspects, including nutrient intake (vitamin B, omega-3 fatty acids, etc.), sugar intake, alcohol consumption, etc. (11–13). Study suggests that adherence to the Mediterranean-Dietary Approaches to Stop Hypertension (DASH) Intervention for neurodegenerative delay (MIND) diet was associated with lower risk of incident dementia in middle-aged and older adults (14–16). Experts estimate that approximately 3% of dementia cases could be prevented through dietary changes. A growing body of literature is reporting the importance of diets for prevention and even slowing down the progression of dementia-related problems (17). In parallel, there is an amplified economic impact due to the growing incidence of dementia, demanding concerted attention from policymakers and healthcare systems.

With bibliometric analysis, a robust quantitative research methodology, it is possible to track the intellectual evolution of a given area of research and its structure in a quantitative way (18). A variety of fields have used it to investigate trends and hotspots in the development of publications (19–21). Although publications relating to diets in MCI and dementia research have steadily increased, it has not yet been possible to conduct a bibliometric analysis. Research concerns identified by such bibliometric studies can be used to guide future research.

Such bibliometric studies can assist investigators in identifying current research concerns and guiding future work. An evaluation of the evolution within this field is intended. This paper examines the diet landscape for people with MCI or dementia by taking into account previous accomplishments, assessing the current state, and predicting the future.

## 2 Materials and methods

### 2.1 Data acquisition

**Figure 1:**
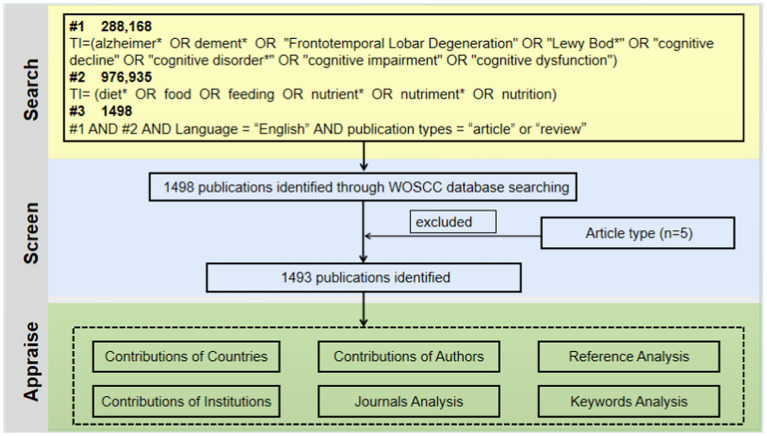
Flowchart of the publications selection.

In order to find publications regarding diet for MCI or dementia, the Web of Science Core Collection (WOSCC) was extensively searched. We employed a seasoned information retrieval expert with over 10 years of experience to oversee and review the development of our search terms. The data search strategy was “TI = Alzheimer* OR dement* OR “Frontotemporal Lobar Degeneration” OR “Lewy Bod*” OR “cognitive decline” OR “cognitive disorder*” OR “cognitive impairment” OR “cognitive dysfunction” AND TI = diet* OR food OR feeding OR nutrient* OR nutriment* OR nutrition AND Language = “English.” This study restricted publication types to “articles” and “reviews” and the search date is November 18, 2023 (Figure 1).

### 2.2 Data analysis and visualization

Downloaded from WOSCC, the data includes fully recorded and cited references. We used three analysis tools such as VOSviewer 1.6.17, CiteSpace 6.1.R3, and R language (version 4.3.0) bibliometrix package (version 4.1.2) for in-depth analysis. Key bibliometric measures include co-authorship, co-citation, and co-occurrence. Co-authorship analysis, for instance, focused on examining collaborative relationships among authors, nations, or institutions, as indicated by jointly authored papers. Analyzing co-occurrences is a quantitative approach to identifying relationships between components. Similarly, co-citation analysis was employed to evaluate the strength of connections between frequently cited elements. Thematic maps and three-field plots were drawn using Bibliometrix, an R package designed for bibliometric analysis.

VOSviewer, a software program for constructing and visualizing bibliometric networks, enabled us to analyze clustering patterns among nations, institutions, authors, and journals based on relationships such as co-authorship, common citations, or direct citations (22). Every node in the map that VOSviewer instantiates represents an element. By considering the quantity of publications, citations, and linkage potency of each component, VOSviewer made it easier to analyze clustering patterns among nations, institutions, authors, and journals. The number of nodes increases with the size of the node, and the number of links between nodes increases as the link width increases. CiteSpace was also a very useful tool for bibliometric analysis at the same time (23, 24).

Using CiteSpace, we analyzed keyword clustering and timelines, as well as the centrality of keywords. In CiteSpace, the following parameters were included: (1) each slice represents a year from 2003 to 2023; (2) single node type selection; (3) G-index based selection criteria, with *k* = 15; (4) using the pathfinder method, pruning was performed. Based on the Journal Citation Reports (JCR) 2022, the impact factor and category quartile data were calculated (25). Researchers, countries, journals, institutions, and journals are evaluated based on the *H*-index, a composite index of academic output.

### 2.3 Research ethics

We did not need Ethics Committee approval since all the data sources we used were publicly available.

## 3 Results

### 3.1 Analysis of publications outputs

**Figure 2:**
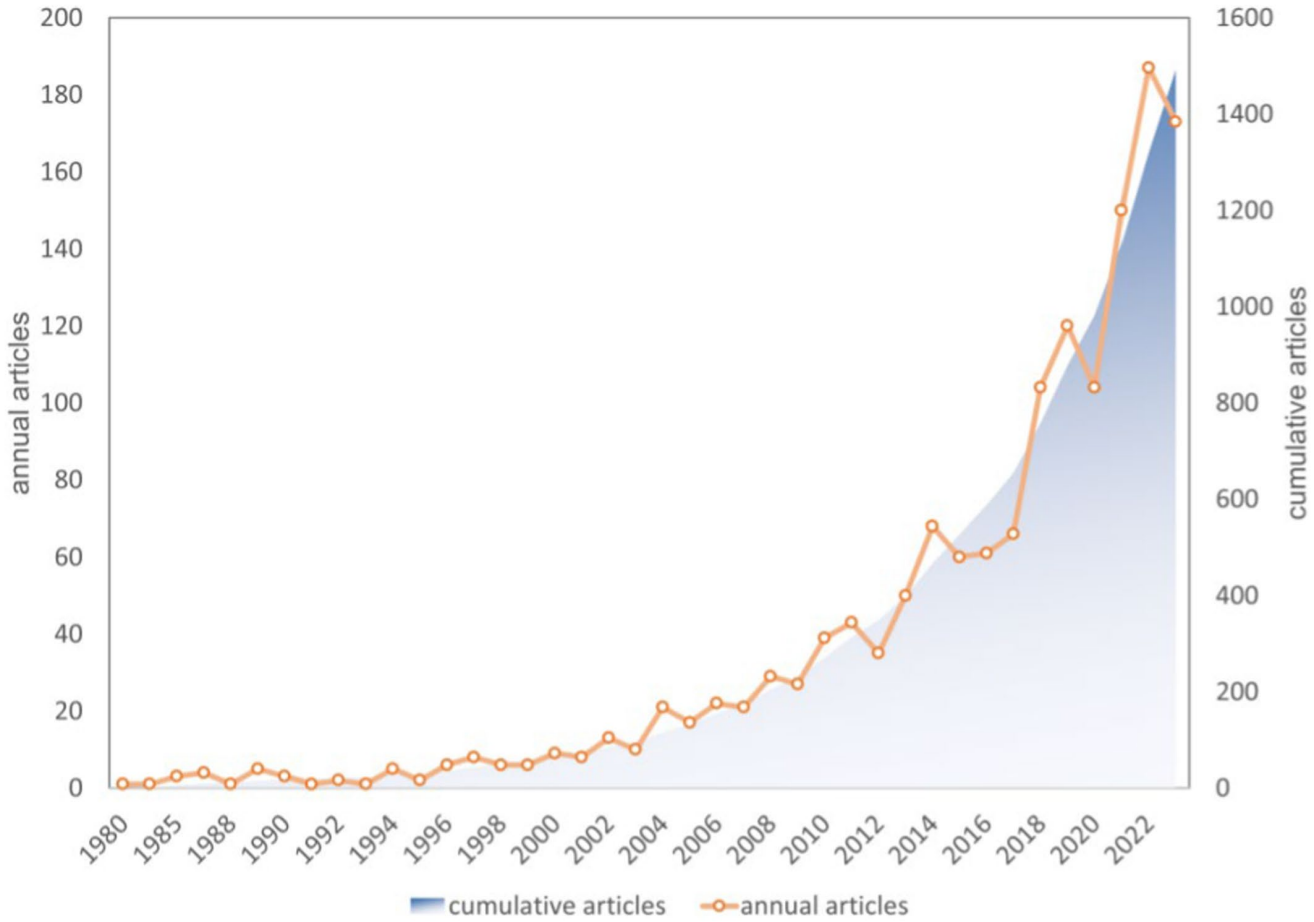
The trend of the annual published articles and cumulative articles.

The diet for MCI and dementia research literature contains 1,493 publications, of which 1,205 articles and 288 reviews account for 80.44 and 19.56%, respectively. Diets for MCI and dementia have been gaining a lot of attention since 2012. Three main phases are involved: (1) slow growth period (1980–2003): a slow and relatively flat growth in the number of annual articles, with the count remaining below 15; (2) stable increase period (2004–2017): in 2004, the annual number of publications exceeded 21 for the first time, and in 2014, 66 publications were published; (3) plateau period (2018–2023): over 100 publications were published annually, a significant increase (Figure 2).

### 3.2 Contribution of countries/institutions

**Figure 3:**
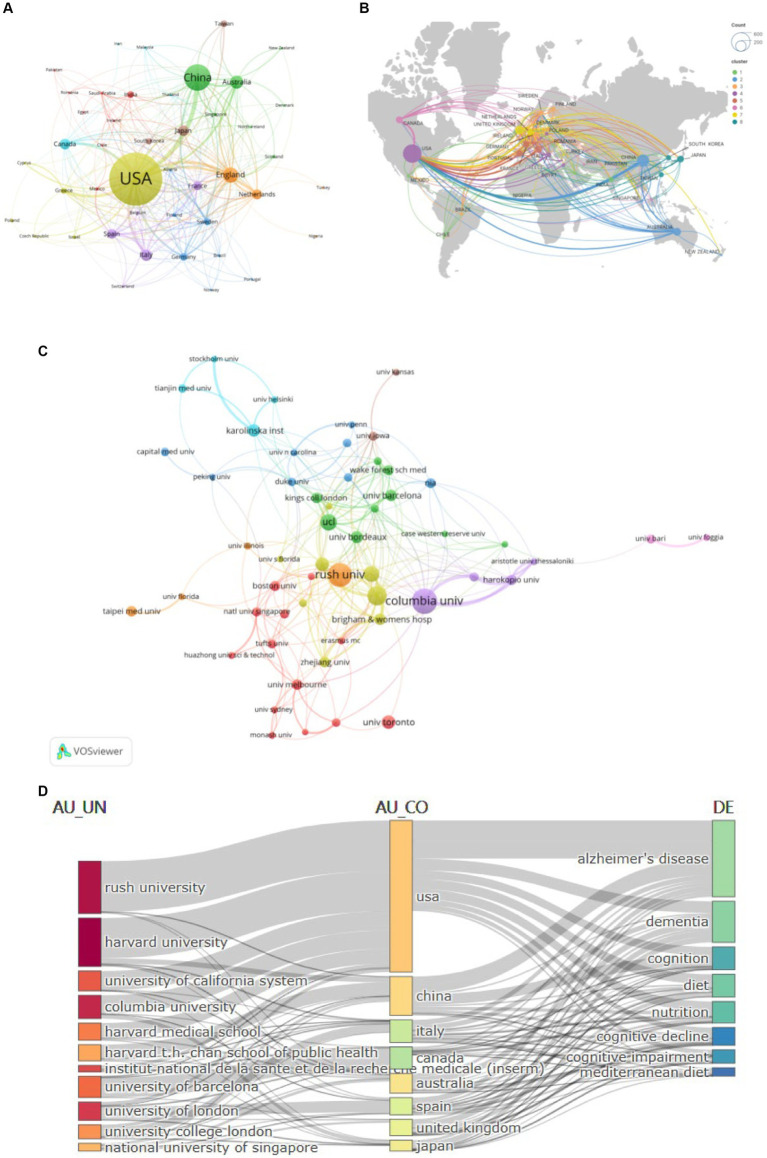
Analysis of Countries/Institutions publications and cooperation. **(A)** Collaboration network analysis of the countries/regions. **(B)** Geographic distribution map. **(C)** Collaboration network analysis of the institutions. **(D)** Three-field plot of affiliations, countries/regions, and keywords.

The diet for MCI and dementia has been published in 82 countries since the turn of the century (Figure 3A). There are 42 countries with cumulative publications greater than five. Among all countries, the USA has published the most publications (557/37.3%), citations (32,950), and *H*-index (94), followed by China (239/16%) and the England (172/11.5%). USA works most closely with other countries (Figure 3B). In addition to Australia, Italy, and Japan, other countries are also showing a growing interest in research. A total of 2,134 institutions are involved in diet for MCI and dementia research (Figure 3C). With 38 cumulative publications and 5,328 citations, Columbia University is the first in the stratum. There are close academic ties, frequent academic interactions, and high-intensity collaborations in the Harvard Medical School, forming a global collaborative network. Figure 3D highlights the country’s collaboration with universities around the world in this field. The density of connections between university names, countries, and research topics gives us a glimpse of the USA leadership in these research fields, as well as the significant global influence and collaborative networks of institutions such as Harvard.

### 3.3 Analysis of authors

**Figure 4:**
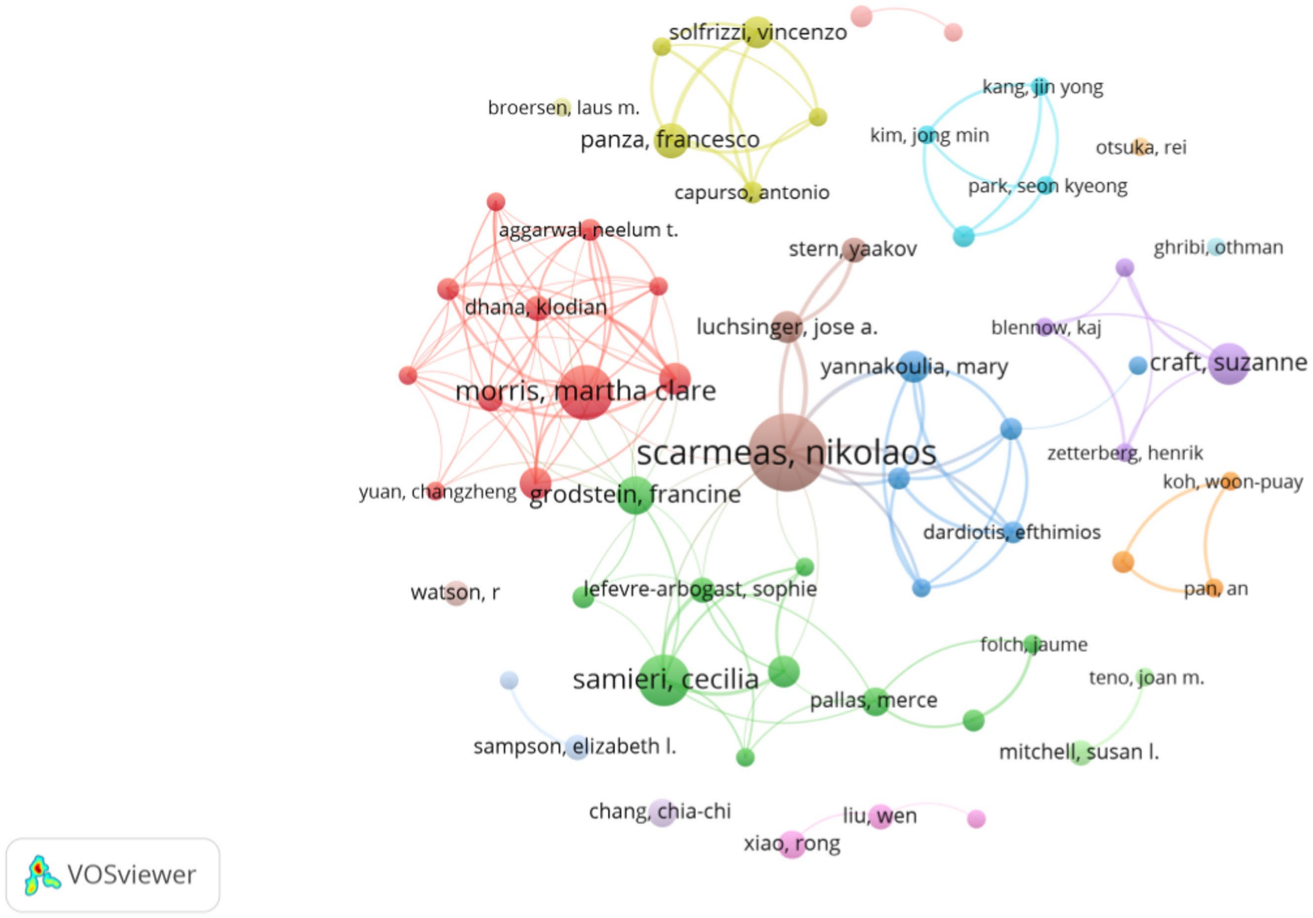
Collaboration network analysis of authors.

In total, 6,819 publications dealt with diet for MCI and dementia were published (Figure 4). Top ten authors are all high impact with H-index over 7 (Top ten authors are with *H*-index over 7), and Scarmeas Nikolaos, Morris Martha Clare, and Samieri Cecilia have 25, 19, and 17 publications, respectively (Table 1). Scarmeas Nikolaos has the highest H-index of over 20 and the highest average citation of 184.6. Martha Clare Morris has the greatest TLS and establishes a tight-knit group.

**Table 1:** Top 10 countries.

Rank	Countries	Counts	Citations	Average citation per paper	TLS	*H*-index	Centrality
1	Brazil	420	6,359	15.13	143	38	0.23
2	USA	382	9,414	24.52	222	49	0.47
3	China	197	2,243	15.12	74	27	0.06
4	England	92	1,682	18.25	121	25	0.35
5	Italy	80	1,299	16.26	49	21	0.16
6	South Korea	77	1,112	14.47	29	19	0.02
7	Australia	72	1,515	15.18	60	22	0.07
8	Canada	70	1967	28.11	40	25	0.13
9	Japan	60	954	7.92	22	9	0.07
10	Germany	52	1,288	25.98	47	19	0.06

TLS, total link strength.

### 3.4 Journals analysis

**Figure 5:**
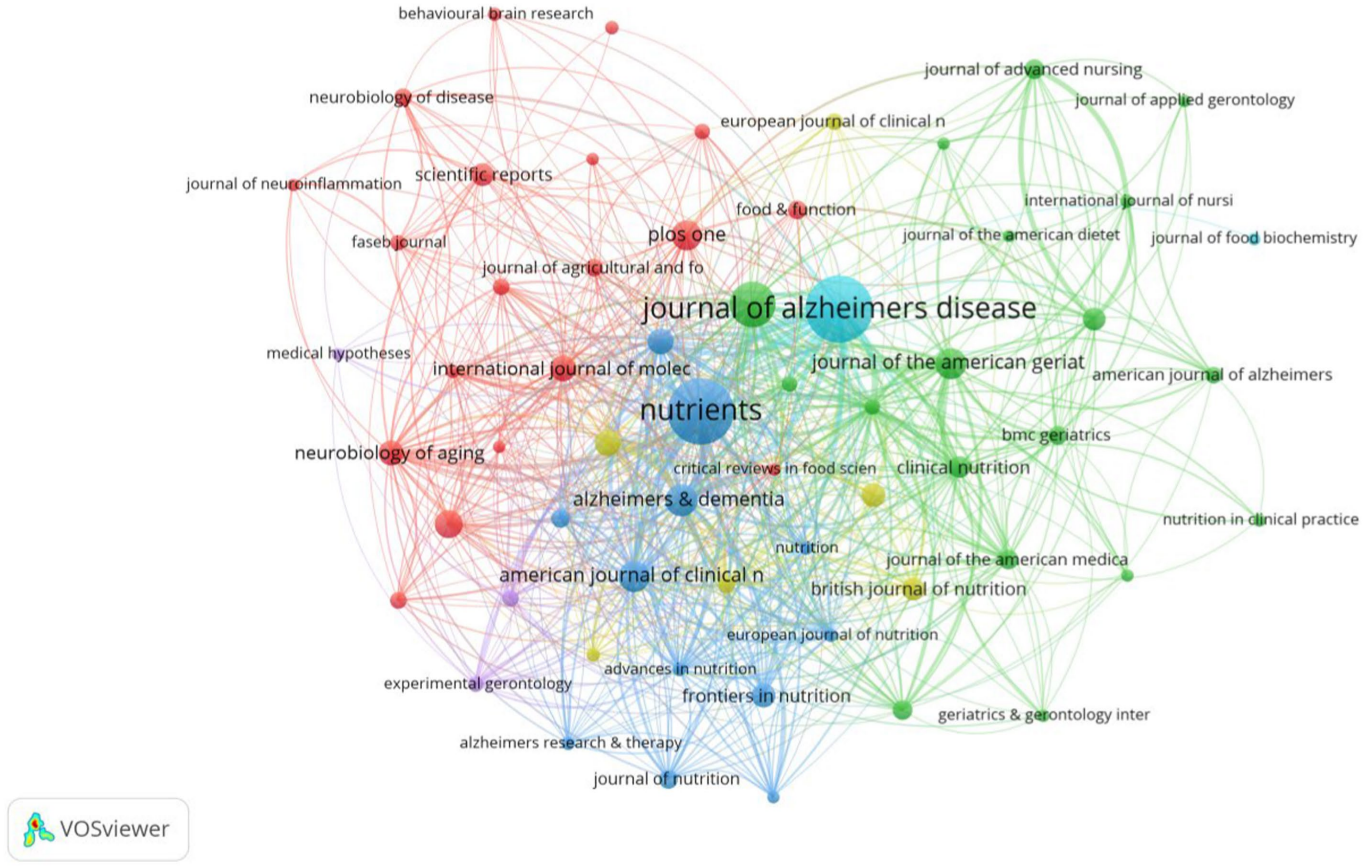
Collaboration network analysis of journals.

There were 521 publications related to diet for MCI and dementia, of which 60 journals had more than six publications (Figure 5). *Journal of Alzheimer’s Disease* topped the list of the 10 most productive journals with 76 publications and 2,911 citations. Followed by the *Journal of Nutrients* and *Journal of Nutrition Health & Aging*, with 75 and 42 relevant publications. It is *Alzheimer’s & Dementia* that has the most impact factor (IF) in the top 10 journals, which confirms their excellent level (Table 2). According to the *H*-index, *Journal of Alzheimer’s disease* (26) and *Journal of Nutrition Health & Aging* (27) have had a profound impact on the development of the field.

**Table 2:** The top 10 productive authors.

Rank	Author	TP	TC	Avg. C	*H*-index	IF(2023)
1	Scarmeas, Nikolaos	25	4,615	184.6	20	Greece
2	Morris, Martha Clare	19	2,445	128.68	16	USA
3	Samieri, Cecilia	17	895	52.65	11	France
4	Panza, Francesco	15	1,492	99.47	13	Italy
5	Craft, Suzanne	14	638	45.57	7	USA
6	Grodstein, Francine	14	1,018	72.71	11	USA
7	Solfrizzi, vincenzo	14	1,480	105.71	13	Italy
8	Bennett, David A.	14	2,283	163.07	10	USA
9	Luchsinger, Jose A. J.	12	3,289	274.08	10	USA
10	Francesco Panza	12	1,275	106.75	12	Italy

TP, total publications; TC, total citations; Avg. C, average citations.

### 3.5 Keywords analysis

**Figure 6:**
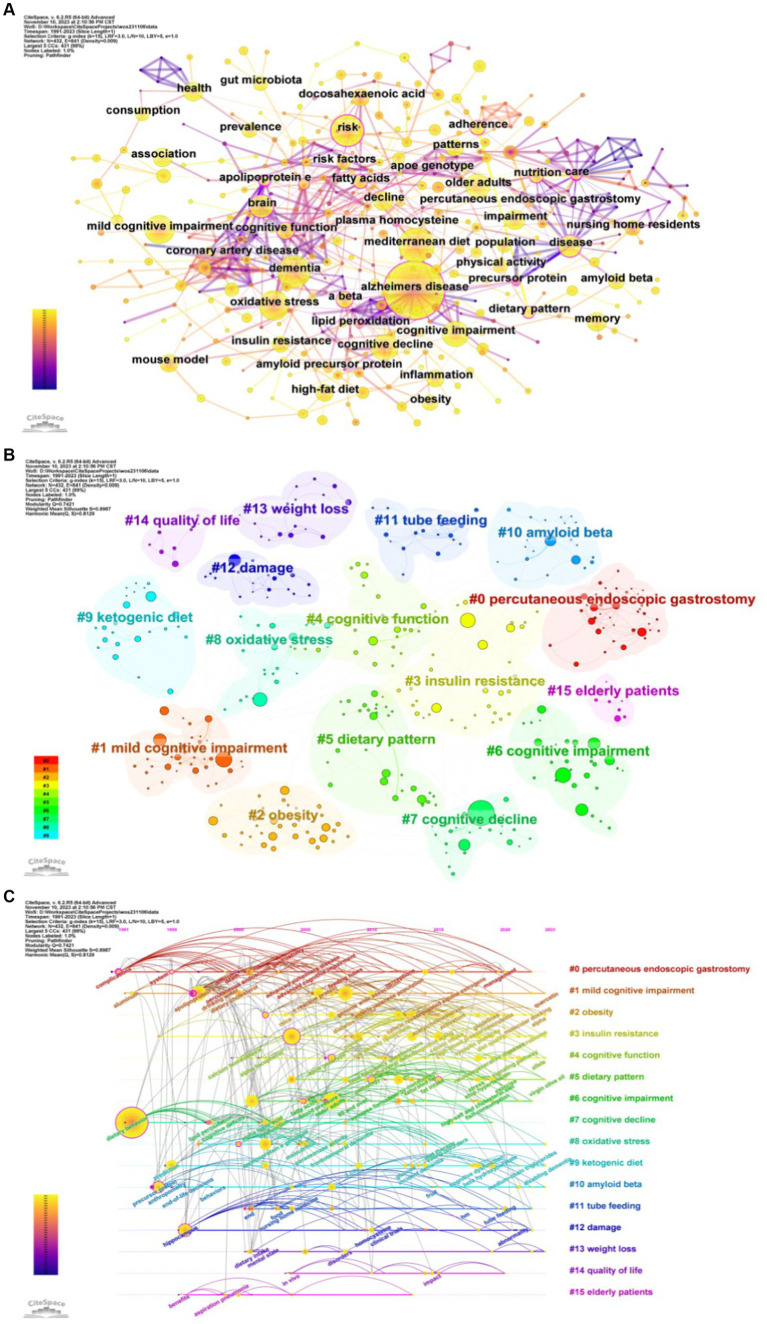
Analysis of keywords. **(A)** Keywords co-occurrence. **(B)** Keyword clustering. **(C)** Timeline of keywords.

The publications yielded 432 keywords, 129 of which appeared more than 10 times (Figure 6A). AD, the name of the disease, ranked highest in frequency (734 occurrences) and association with other keywords. In addition, the top five frequency keywords also include dementia, Mediterranean diet, risk, oxidative stress. The top three centrality are apolipoprotein E, care, amyloid beta (Table 3). Using clustering analysis of keywords, we found 16 cross-over clusters, mostly related to disease name and classification (#1, #4, #6, #7), dietary patterns and specific nutritional patterns (#5, #9, #11, #0), Physiological factors and health status (#2, #3, #8, #10, #13), quality of life and health consequences (#12, #14, #15) (Figure 6B). Timeline plots were used to display temporal trends (Figure 6C). In the field of diet for MCI and dementia, “#1 mild cognitive impairment,” “#2 obesity,” “#3 insulin resistance,” “#4 cognitive function,” “#5 dietary pattern,” “#8 oxidative stress,” “#9 Ketogenic diet (KD),” “#10 amyloid beta” and “#13 weight loss” emerge as recent research frontiers.

**Table 3:** Top 10 journals in the field of exercise for hypertension.

Rank	Journal	TP	TC	Avg. C	*H*-index	IF (2023)
1	Journal of Alzheimer’s Disease	76	2,911	38.3	36	4.0/Q2
2	Nutrients	75	1,106	14.75	21	5.9/Q1
3	Journal of Nutrition Health & Aging	42	1,368	32.57	23	5.8/Q2
4	Alzheimer’s & Dementia	25	1806	72.24	15	14.0/Q1
5	Journal of the American Geriatrics Society	24	1,447	60.29	21	6.3/Q1
6	American Journal of Clinical Nutrition	23	1,181	51.35	14	7.1/Q1
7	PLoS One	22	582	26.45	13	3.7/Q2
8	Frontiers in Aging Neuroscience	20	391	19.55	11	4.8/Q1
9	Current Alzheimer Research	18	674	37.44	11	2.1/Q3
10	International Journal of Molecular Sciences	18	440	22.44	9	5.6/Q1
11	Neurology	18	1,618	89.88	13	9.9/Q1

TP, total publications; TC, total citations; Avg. C, average citation.

**Figure 7:**
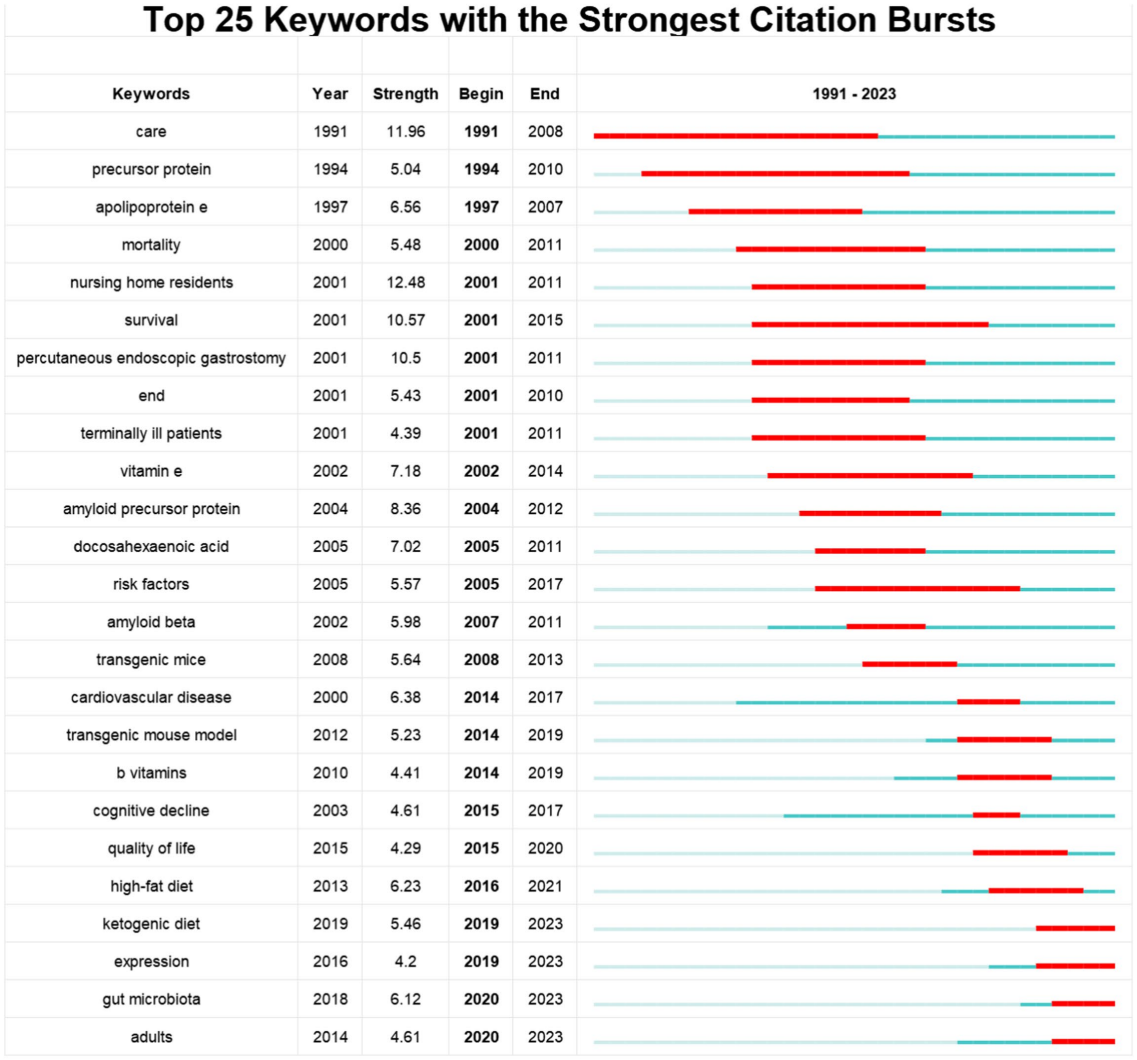
Top 25 keywords with the strongest citation bursts.

Using CiteSpace software, the top 25 keywords on diet for MCI and dementia are analyzed to determine the hotspots and research trends (Figure 7). This timeline analysis of burst keywords specific to diet and dementia reveals a chronological progression of research focus areas. Initial bursts of keywords like “care” (1991–2008), “nursing home residents” (2001–2011), and “survival” (2001–2015), highlight an early emphasis on the practical aspects of care and longevity in dementia patients. Furthermore, the prominence of “percutaneous endoscopic gastrostomy” (2001–2011) underscores a period where nutritional interventions via medical procedures were a key area of investigation. “Ketogenic diet” has gained popularity, as well as “expression” and “gut microbiota.” Diet research for MCI and dementia is currently garnering attention, and indicates potential developments in the field.

### 3.6 Reference analysis

**Figure 8:**
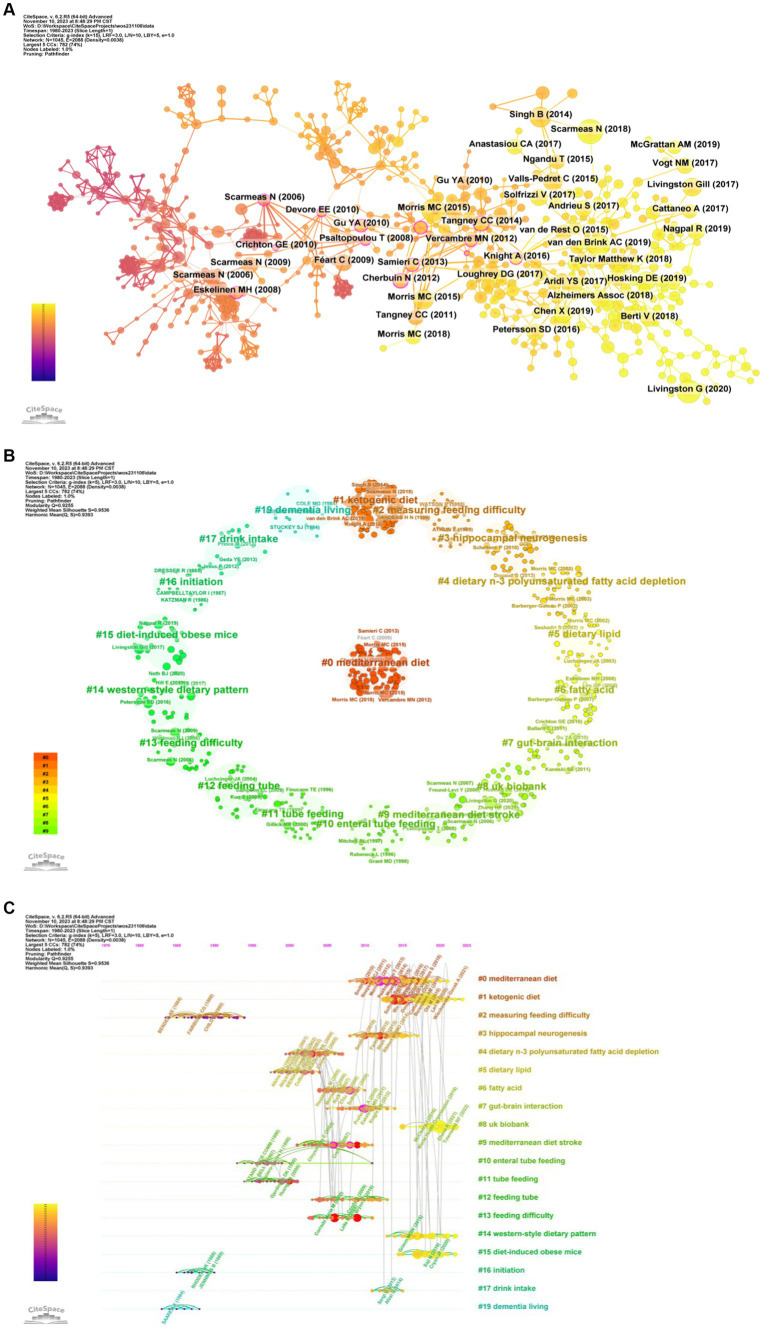
Analysis of references. **(A)** References co-occurrence. **(B)** References clustering. **(C)** Timeline of references.

We extracted 33,350 references, of which 872 were cited more than 10 times (Figure 8A). The top 10 cited references are mainly articles, focusing on the correlation between dietary patterns and dementia diseases. These important studies have been predominantly published in journals of significant influence, such as *Lancet Neurol*, *Lancet*, and *Alzheimer’s Dement* (Table 4). The most frequently cited reference is a review, which published in the *Lancet Neurol* written by Scarmeas N. harvested 62 citations. The paper by Livingston G. et al. was ranked second with 55 citations and there were 49 citations for the paper by Morris M. C. et al. Three of the most cited key papers emphasize the significant impact dietary and lifestyle interventions can have on reducing cognitive impairment, dementia, and AD (11, 28, 29). These studies reveal that specific nutrients and diets, notably the MIND diet, are associated with a marked decrease in AD incidence, emphasizing the role of modifiable factors such as nutrition, education, and lifestyle changes in dementia prevention (see Table 5).

**Table 4:** Top 20 keywords in the field of diet for MCI and dementia.

Rank	Keywords	Occurrences	Centrality	Rank	Keywords	Occurrences	Centrality
1	Alzheimer’s disease	734	0.17	11	insulin resistance	92	0.05
2	dementia	265	0.08	12	impairment	91	0.01
3	Mediterranean diet	247	0.06	13	cognitive function	83	0.18
4	risk	224	0.16	14	memory	77	0.04
5	oxidative stress	219	0.06	15	mouse model	73	0.05
6	mild cognitive impairment	173	0.01	16	nursing home residents	72	0.03
7	brain	160	0.16	17	amyloid beta	72	0.03
8	cognitive impairment	158	0.05	18	docosahexaenoic acid	71	0.03
9	older adults	149	0.02	19	high-fat diet	70	0.02
10	cognitive decline	142	0.04	20	inflammation	68	0.02

**Table 5:** Top 10 references in the field of diet for MCI and dementia.

Rank	References	Co-cited counts	JCR quartile	IF (2023)
1	Scarmeas N., Anastasiou C. A., Yannakoulia M. Nutrition and prevention of cognitive impairment. *Lancet Neurol*. (2018). 11. 1006–1015	62	Q1	48
2	Livingston G., Huntley J., Sommerlad A. et al. Dementia prevention, intervention, and care: 2020 report of the Lancet Commission. *Lancet*. (2020). 396. 413–446	55	Q1	168.9
3	Morris M. C., Tangney C. C., Wang Y. et al. MIND diet associated with reduced incidence of Alzheimer’s disease. *Alzheimers Dement*. (2015). 9. 1007–1014	49	Q1	14
4	van den Brink A. C., Brouwer-Brolsma E. M., Berendsen A. M. et al. The Mediterranean, Dietary Approaches to Stop Hypertension (DASH), and Mediterranean-DASH Intervention for Neurodegenerative Delay (MIND) diets are associated with less cognitive decline and a lower risk of Alzheimer’s disease-a review. *Adv Nutr*. (2019). 6. 1040–1065	45	Q1	9.3
5	Singh B., Parsaik A. K., Mielke M. M. et al. Association of mediterranean diet with mild cognitive impairment and Alzheimer’s disease: a systematic review and meta-analysis. *J Alzheimers Dis*. (2014). 2. 271–282	43	Q2	4.0
6	Morris M. C., Tangney C. C., Wang Y. et al. MIND diet slows cognitive decline with aging. *Alzheimers Dement*. (2015). 9. 1015–1022	40	Q1	14
7	Hosking D. E., Eramudugolla R., Cherbuin N. et al. MIND not Mediterranean diet related to 12-year incidence of cognitive impairment in an Australian longitudinal cohort study. *Alzheimers Dement*. (2019). 4. 581–589	37	Q1	14
8	Livingston G., Sommerlad A., Orgeta V. et al. Dementia prevention, intervention, and care. *Lancet*. (2017). 390. 2673–2734	37	Q1	168.9
9	Valls-Pedret C., Sala-Vila A., Serra-Mir M. et al. Mediterranean diet and age-related cognitive decline: a randomized clinical trial. *JAMA Intern Med*. (2015). 7. 1094–1103	36	Q1	39
10	Alzheimer’s Association. Alzheimer’s disease facts and figures. *Alzheimers Dement*. (2018). 14. 701	34	Q1	14

**Figure 9:**
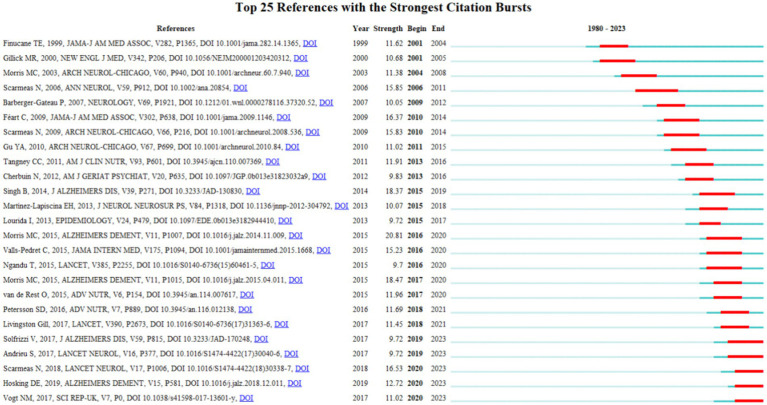
Top 25 references with the strongest citation bursts.

A clustering analysis of references reveals that most of the references relate to dietary patterns and specific nutrients (#0, #1, #4, #5, #6#14), cognitive impairment is associated with disease (#3, #7, #9, #15, #19), feeding methods and related challenges (#2, #10, #11, #12, #13, #17), research data resources (#8, #16) (Figures 8B,C). Meanwhile, the burst analysis displayed the top 25 references (Figure 9). The five latest bursts and continuously emerging references emphasis the importance of diet in the research of cognitive impairment and dementia, particularly AD. These studies collectively highlight significant insights into the gut-brain axis, the effectiveness of specific dietary patterns like the MIND diet in reducing AD incidence, and the broader impact of nutrition on cognitive health. They reveal key associations between gut microbiota diversity, dietary patterns, and cognitive outcomes, advocating for a multifaceted approach in understanding and managing cognitive disorders. Although diet has a potentially positive impact on cognitive health in older adults, more research is needed to confirm it (29–33).

### 3.7 Analysis of topic trends

**Figure 10:**
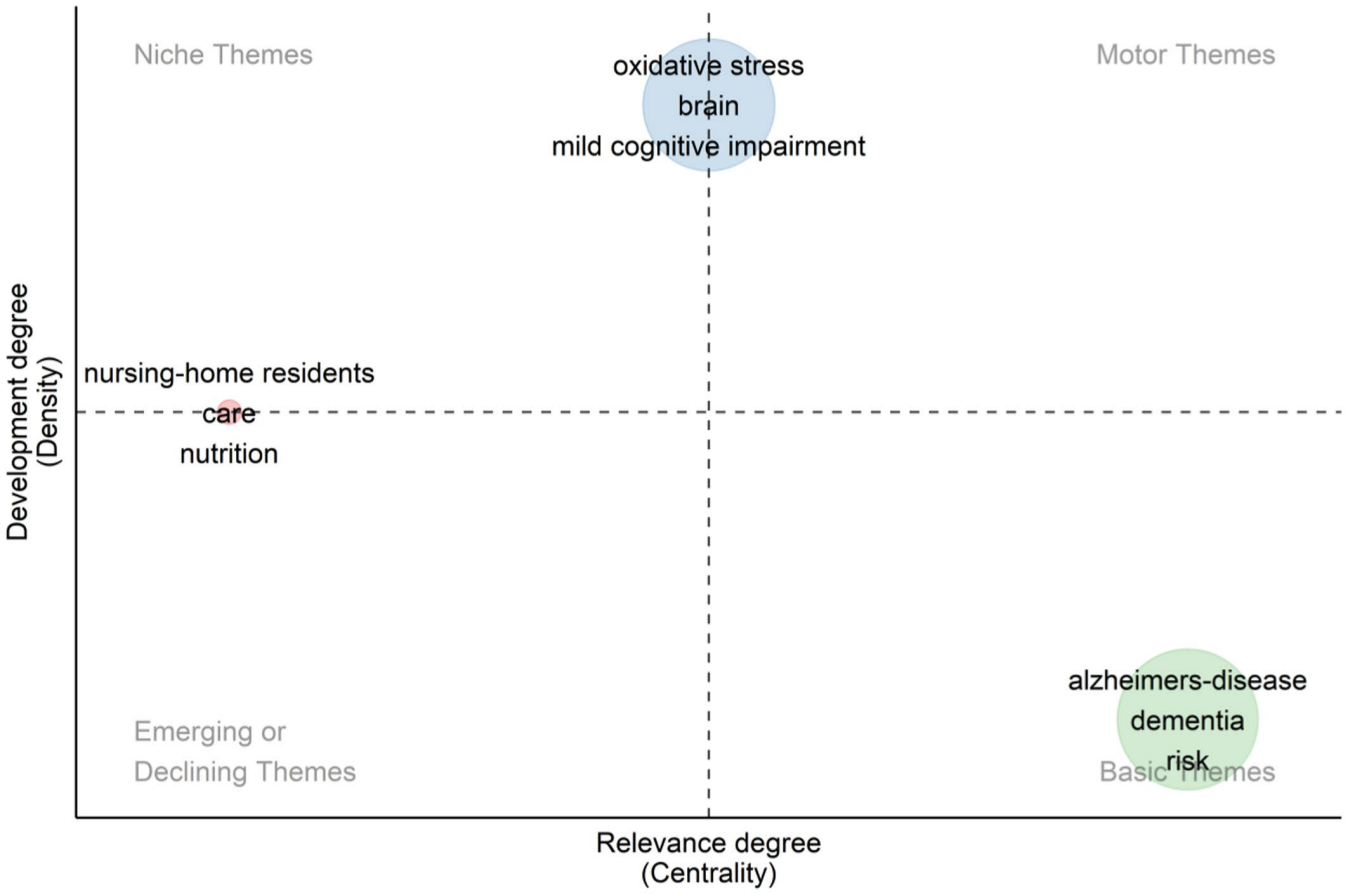
Thematic map.

From the Figure 10, we can identify different categories of research topics in the fields of diet, cognitive impairment, and dementia. The figure shows “Alzheimer’s-disease” and “dementia” as the basic themes, which occupy a central position in the field of research, showing their status as core research topics. In addition, the placement of topics such as “Oxidative Stress,” “Brain” and “Mild Cognitive Impairment” indicates that they are currently actively developing research areas, reflecting the dynamics and trends of current scientific research. These developing topics indicate new research opportunities, as well as possible future research directions. Conversely, topics in the upper left and lower left quadrants may indicate a declining research interest.

## 4 Discussion

Throughout the world, MCI and dementia have increased significantly, causing a huge medical and social burden. At present, there is no effective drug treatment regimen of MCI and dementia (34). Public and academic researchers are looking for ways to prevent and treat diseases based on lifestyle factors. Studies have shown that a sensible diet has a good effect on alleviating cognitive decline. The main dietary patterns include MIND diet, DASH diet, Mediterranean diet and KD (27, 35, 36). In order to prevent and treat MCI and dementia, diet is expected to become a key aspect of prevention and treatment.

### 4.1 General information

Using the WoSCC databases, we searched for articles published about diet and MCI in the present study. The bibliometric study consisted of 1,493 papers from 2,134 institutions in 82 countries and regions with 6,819 authors, published in 521 journals with 33,350 co-cited references. Research on diet for MCI and dementia has experienced a slow growth period (1980–2003), a stable increase period (2004–2017), and may now be at an academic plateau. Recent years have seen an increase in the amount of scientific research produced on diet for MCI and dementia.

### 4.2 Research hotspots

Cognitive impairment is a broad term that usually refers to a slight decline in memory, thinking, and judgment. MCI was first proposed by a team from New York University in the 80s of the 19th century as a form of cognitive impairment and is often seen as the primary stage of cognitive decline (37). On beginning of MCI, memory and cognitive abilities may decline further and may progress to more severe dementia. Dementia is an umbrella term that encompasses a variety of conditions with severe cognitive decline, the most common of which is AD, which is characterized by memory loss and loss of thinking and language skills (38). There are other types of dementia, such as vascular dementia, dementia with Lewy bodies, and dementia in the frontotemporal lobe, each with different causes and manifestations (39). It is important to note that not all people with mild cognitive impairment will develop dementia. Diagnosis and early intervention are important to manage and improve these conditions.

When exploring the management of cognitive impairment and dementia, it is particularly noteworthy that the KD, a dietary style that has attracted a lot of attention in cognitive impairment research in recent years. The KD, which refers to a high-fat, low-carbohydrate diet, has attracted a lot of attention in recent years in the study of cognitive impairment (40, 41). This eating pattern is designed to guide the body into ketosis, allowing the brain to use ketones instead of glucose as the primary energy source. This may be important for people with cognitive impairment, especially AD, who often have impaired ability to metabolize glucose in the brain. However, the strictness of the KD and the potential risk of nutritional imbalance are also factors to consider (42). Other dietary patterns are currently being studied in the MIND diet, DASH diet, and Mediterranean diet, which mainly protect the brain from inflammation and oxidative damage and reduce the risk of cognitive impairment by advocating more fruits, reducing the intake of fried foods, and improving the body’s antioxidant and anti-inflammatory properties (16, 27, 41). In addition to exploring different dietary patterns, such as the KD, the study also focused on dietary solutions for patients who were unable to eat on their own due to cognitive decline. In this case, tube feeding and percutaneous endoscopic gastrostomy (PEG) are important options to consider. Tube feeding refers to the intubation of the stomach through the nose, and PEG refers to the entry of the stomach through a small incision in the abdomen (26). Patients with dementia often have difficulty eating and drinking, and for people with dementia who are unable to eat on their own, tube feeding or PEG can provide essential nutrients (43). However, tube feeding has side effects such as aspiration, gastric retention, and the risk of accidental extubation, while PEG can be painful and may cause other adverse effects (pneumonia, poor bowel or bladder control, and bleeding, swelling, and infection), and its use is accompanied by ethical and clinical management challenges (26).

In recent years, studies have found that obesity, insulin resistance, oxidative stress, amyloid beta, and weight changes play an important role in cognitive decline. Chronic inflammatory states and metabolic disorders due to obesity can negatively impact brain health, including affecting cognitive function and promoting the development of neurodegenerative diseases (44). At the same time, obesity is associated with an increased risk of cardiovascular disease, which is also considered to be an important factor affecting cognitive impairment (45). In the brain, insulin not only regulates blood sugar levels, but is also involved in neuronal growth, survival, and synaptic function. Insulin resistance can cause neuronal damage and reduce synaptic plasticity, which can affect memory and learning ability (46). Moreover, insulin resistance is associated with abnormal accumulation of AD biomarkers such as amyloid beta and tau protein (47). Amyloid beta is the main component of age plaques in AD (48). Numerous studies have shown that the accumulation of amyloid beta is thought to be a key process leading to nerve cell damage and death, thereby affecting cognitive function. Accumulation in both nerve cells and outside of nerve cells can cause toxic reactions, leading to neuronal degeneration and death (48, 49). Clearance of neurotoxic amyloid beta in the brain is the primary strategy for remission and treatment of AD (50). Oxidative stress is a state of cellular damage caused by excessive production of free radicals, which plays an important role in the pathogenesis of cognitive impairment such as AD, especially related to the abnormal deposition of amyloid beta (51).

A large number of studies have focused on genetic factors closely related to MCI or AD, especially the gene polymorphisms of apolipoprotein E (ApoE). In addition to its function as a polymorphic protein, ApoE genes can be involved in many biological processes, which may contribute to the pathogenesis of dementia, particularly AD (52). ApoE has three gene variants, ε2, ε3, and ε4. In 1997, Farrer L. A. found that the frequency of ε4 increased in patients with late-onset familial AD. Subsequently, 46.2% of AD patients were found to carry the ε4 allele, while 13.2% of the control group were carried. In 2002, Zubenko et al. (53) reported that centenarians generally carry the ε2 gene. This discovery is a big step forward for ApoE polymorphisms and research. The relationship between ApoE polymorphisms and AD has been mainly focused on brain metabolism related to amyloid beta or tau protein, as well as neuronal growth and branching. Research on ApoE and its gene polymorphisms has become a hot topic in medicine. Considering the role of ApoE in the metabolism of lipoproteins, adjusting the type and amount of fat in the diet may be particularly important for ε4 carriers. At the same time, studies have found that nursing has been paid more and more attention in recent years in the direction of dietary intervention for MCI and dementia, and has achieved certain results (52, 54, 55).

The top three most cited key papers highlight the importance of diet in preventing cognitive impairment and dementia, especially AD. The study by Nikolaos Scarmeas published in 2018 highlighted nutrition as a lifestyle intervention that can alter cognitive decline risks (11). Despite the limited evidence, the study notes there is a protective association between certain nutrients (e.g., folate, flavonoids, vitamin D, and certain lipids) or food groups (e.g., seafood, vegetables, and fruits, and potentially moderate alcohol and caffeine consumption) and cognitive outcomes among older adults. Moreover, According to Livingston’s et al. (16) article, the incidence of dementia has fallen in many countries due to improvements in education, nutrition, health care, and lifestyle changes. Together the 12 modifiable risk factors (smoking, obesity, depression and et al.) as a result, around 40% of dementia cases worldwide may be prevented or delayed. In low-and middle-income countries where dementia occurs more frequently, prevention potential is higher. Morris MC, et al. published an article titled MIND diet associated with reduced incidence of AD in 2015 (29). The study found that the MIND diet was associated with a significant reduction in the risk of AD, with lower prevalence with higher adherence, with a 53% reduction in incidence in the group with the highest adherence, and even moderate adherence to the MIND diet may have significant benefits in preventing AD. The three studies focused on the role of diet in preventing cognitive impairment and dementia, highlighting the association of specific nutrients and healthy eating patterns such as the MIND diet with a reduced risk of AD. The study also identified several modifiable risk factors, including education, lifestyle, and environmental factors.

### 4.3 Feature trends

Three new trends in diet for MCI and dementia research were identified by analyzing keyword references with citation bursts. These are:

**The Ketogenic diet (KD)**: first proposed by Can Med Assoc J in 1931 for managing epilepsy, particularly in children with refractory seizures, has since expanded its therapeutic potential to include the treatment of neurodegenerative diseases like AD (56). Recent studies suggest that the KD, by upregulating circulating β-hydroxybutyrate and altering gut microbiome composition and neurovascular functions, offers neuroprotective effects (57). These benefits stem from its ability to reduce oxidative stress at the mitochondrial level through ketone and polyunsaturated fatty acid (PUFA) production. Oxidative stress may also be a future research direction for the prevention and treatment of cognitive impairment (58). Additionally, evidence indicates the KD’s efficacy in reducing midlife mortality rates and improving memory in mice.

**Expression**: since 2016, there is a significant peak in citations of the keyword “expression,” indicating a growing scientific interest in gene or protein expression patterns related diet, cognitive impairment, and the development of dementia symptoms. Key focus areas included the interaction between diet and gene expression, especially antioxidants and omega-3 fatty acids have been found to influence cognitive health by regulating gene expression (59). The ε4 allele in the APoE gene is an important genetic marker for AD risk, and research focuses on how this gene interacts with dietary factors and influences the development of cognitive impairment (55). In addition, epigenetic studies revealed how environmental factors, especially diet, alter gene expression through mechanisms such as DNA methylation and histone modifications, which in turn influence the development of neurodegenerative diseases (60). Protein expression changes are also crucial in cognitive impairment development, with dietary factors affecting the expression of proteins associated with AD (61). Furthermore, “expression” is not only reflected in the expression of genes and proteins at the biological level, but also involves the expression of patients’ emotions and thoughts (62).

**Gut microbiota**: recent neuroscience research has focused on the interaction between gut microbiota and brain function, or the “gut-brain axis.” Intestinal flora plays a variety of roles in the occurrence and development of AD, according to recent studies. Thus, intestinal flora modulation is now considered a new treatment option for AD (63). By affecting the nervous, endocrine, metabolic, and immune systems, the intestinal flora affects AD occurrence primarily (64). AD has recently been treated and prevented by regulating intestinal flora (65). A study found that AD-associated intestinal flora (characterized by Bacteroidetes) enrichment could enhance microglial cell activation and neuroinflammation by activating the C/EBPβ/AEP pathway and upregulating pro-inflammatory PUFA metabolism by activating the C/EBPβ/AEP pathway, thereby promoting microglial cell activation and neuroinflammation, thereby promoting AD pathology and cognitive impairment. AD patients had different intestinal flora than healthy volunteers and patients with MCI (66).

Emerging analysis highlighted five key papers as the latest hotspots in the research on diet for cognitive impairment/dementia. Nicholas’s M. study unearthed significant differences in the gut microbiome of AD patients, notably a decrease in microbial diversity and distinct variations from age-and sex-matched controls, including diminished Firmicutes and elevated Bacteroidetes. This study also discovered correlations between various microbial genera and AD biomarkers in cerebrospinal fluid, suggesting a possible link for therapeutic intervention (33). In addition to the exploration of the gut-brain axis, another significant stride in this field is Martha’s C. M. research on the MIND diet, which has shown promising results in reducing AD incidence. Over a 4.5 year period, the study followed 923 participants aged 58–98, demonstrated the effectiveness of the MIND diet in lowering the rates of AD. Compared with DASH and Mediterranean diets, higher adherence to the MIND diet significantly reduced AD rates (29). Similarly, Diane’s E. Australian longitudinal study reinforced the MIND diet’s effectiveness, showing a reduced 12-year incidence of cognitive impairment, contrasting with the Mediterranean diet, which did not exhibit the same level of effectiveness (30). Scarmea’s et al. (31) research delved into the broader spectrum of nutrition and its preventive role against cognitive impairment, highlighting a protective association between certain nutrients and dietary patterns, especially the Mediterranean diet, and cognitive outcomes in the elderly. Lastly, Vincenzo’s systematic review synthesized recent observational studies, revealing a direct relationship between dietary patterns and brain structure/activity changes. It highlighted the synergistic health effects of dietary patterns like the Mediterranean, DASH, and MIND diets in reducing cognitive decline and AD rates (32). These comprehensive studies collectively underscore the critical role of diet in cognitive health, ranging from microbial interactions to dietary patterns, and advocate for a multifaceted approach to managing and understanding cognitive disorders.

### 4.4 Limitation

In comparison to traditional reviews, bibliometric-based analyses provide a broader perspective on research priorities and trends in diet for MCI and dementia.

But the research has its limitations. Firstly, due to the continuous updating of WoSCC, the retrieval results of this study may not reflect the most recent literature. Secondly, no other databases were searched, such as Google Scholar or PubMed. Thirdly, this analysis might be less credible if the literature collected is of uneven quality. Lastly, only English literature is included, and some important non-English publications may be excluded, leading to the neglect of non-English papers.

Furthermore, the inherent challenges of dietary pattern research, particularly in relation to MCI and dementia, due to a myriad of confounding factors, compound the difficulty in drawing definitive conclusions. This complexity is exacerbated in a bibliometric context, where the reliance on secondary literature analysis navigates compounded uncertainties and may not fully capture the nuanced effects of dietary factors on cognitive health. The quality and biases inherent in the existing literature, including a possible publication bias towards positive findings, further limit the conclusions drawn from our analysis. By addressing these limitations, our aim is to present a comprehensive, transparent account of our study’s scope and the potential impacts of our findings, thereby not only enhancing the credibility of our research but also pointing to areas for future methodological refinement and investigation.

## 5 Conclusion

Plenty of studies about diet and MCI and dementia have been conducted in recent years, especially since 2018. As a result, Columbia University and the United States have a very strong presence in this field. The most productive journal was *Journal of Alzheimer’s Disease*. In addition, articles, authors, and institutions that have greatly impacted this field were identified by the study. Current research shows that keywords such as “Ketogenic diet,” “expression,” “APoE”, and “gut microbiota” may represent the latest research hotspots and frontiers. Researchers gained new insight into future research directions and scientific decisions as a result of the study.

## Author contributions

YL: Conceptualization, Data curation, Formal analysis, Funding acquisition, Investigation, Methodology, Project administration, Resources, Software, Supervision, Validation, Visualization, Writing – original draft, Writing – review & editing. XC: Methodology, Software, Writing – review & editing. LeZ: Methodology, Visualization, Writing – review & editing. NX: Software, Visualization, Writing – review & editing. LiZ: Supervision, Writing – review & editing. WH: Visualization, Writing – review & editing. YQ: Supervision, Writing – review & editing.
